# Detection and Characterization of Circulating Tumour Cells in Multiple Myeloma

**DOI:** 10.5772/64124

**Published:** 2016-01-01

**Authors:** Liangxuan Zhang, Sharon Beasley, Natalie L. Prigozhina, Renee Higgins, Shoji Ikeda, Florence Y. Lee, Dena Marrinucci, Shidong Jia

**Affiliations:** 1 Departments of Oncology Biomarker Development, Genentech Inc, South San Francisco, CA, USA; 2 Epic Sciences Inc., San Diego, CA, USA; 3 Predicine Inc, Hayward, CA, USA

**Keywords:** Circulating Tumour Cells, Rare Cells, Liquid Biopsy, Multiple Myeloma, Biomarkers, Peripheral Blood, Drug Development

## Abstract

Multiple myeloma (MM) remains an incurable disease despite recent therapeutic improvements. The ability to detect and characterize MM circulating tumour cells (CTCs) in peripheral blood provides an alternative to replace or augment invasive bone marrow (BM) biopsies with a simple blood draw, providing real-time, clinically relevant information leading to improved disease management and therapy selection. Here we have developed and qualified an enrichment-free, cell-based immunofluorescence MM CTC assay that utilizes an automated digital pathology algorithm to distinguish MM CTCs from white blood cells (WBCs) on the basis of CD138 and CD45 expression levels, as well as a number of morphological parameters. These MM CTCs were further characterized for expression of phospho-ribosomal protein S6 (pS6) as a readout for PI3K/AKT pathway activation. Clinical feasibility of the assay was established by testing blood samples from a small cohort of patients, where we detected populations of both CD138^pos^ and CD138^neg^ MM CTCs. *In* this study, we developed an immunofluorescent cell-based assay to detect and characterize CTCs in MM.

## 1. Introduction

Multiple myeloma (MM) is a neoplasm of plasma cells and is the second most common blood malignancy worldwide [1], accounting for 1% of all cancers, 13% of hematologic malignancies [2], and causing approximately 20% of hematologic malignancy-related deaths [3]. Though MM is still incurable, in the last decade patients with MM have experienced an increased overall survival (up to eight years) due to considerable improvements in disease management, disease monitoring, and the introduction of several new therapeutics, including bortezomib, lenalidomide and thalidomide [1, 2, 4–9].

A number of signalling pathways are known to be dysregulated in MM, which contributes to disease progression. In particular, aberrant activation of growth factor pathways results in downstream activation of the PI3K/AKT signalling cascade, promoting cell proliferation, survival and tumour growth [10, 11]. Targeting the PI3K/AKT pathway in MM has been shown to induce cell-cycle arrest and apoptosis in MM cell lines and patient myeloma cells [12–14]. Multiple clinical trials involving the use of PI3K/AKT pathway inhibitors to treat relapsed or refractory MM are underway ([10, 15], clinicaltrials.org).

Further improvements in MM treatment will require development of novel targeted therapies in combination with diagnostic tests, as well as sensitive technologies to monitor residual disease in patients during remission. Since cancer is a heterogeneous and dynamic disease as demonstrated by inter-patient variability [16, 17] and intra-patient evolution over time [17–22], the management of patients on targeted therapies requires detailed and up-to-date molecular roadmaps, for which repeated and non-invasive sampling will be crucial [23, 24]. Current standard of care requires invasive and painful bone marrow (BM) biopsies, which are not ideal for routine observation of patients' progress in real time. Thus, the development of a liquid (blood) biopsy for detection and molecular characterization of MM tumour cells in peripheral blood could have broad clinical utility and improve patients' quality of life.

While originating in the BM, MM tumour cells are able to migrate into the peripheral blood stream, from where they can be isolated and characterized ([25] and references therein). These MM circulating tumour cells (CTCs) have been used as biomarkers to indicate active disease [26], to assess disease stage [27], to stratify MM patients for autologous stem-cells transplantation [28, 29], to predict survival [30] and to monitor response to therapy [31]. While flow cytometry is the most commonly used method to analyse MM CTCs, the sensitivity of this technology remains relatively low. Different groups using flow cytometry report varying numbers of patients with detectable CTCs ranging from 30–75% [27, 29, 32–34]. In addition, for patients with MM CTCs, the numbers of MM CTCs identified in peripheral blood were generally low [27, 33]. As an alternative to flow cytometry methods, Cellsearch® technology, which utilizes magnetic particles coated with anti-CD138 (syndecan-1) antibodies to enrich MM CTCs from the blood, has been explored for its ability to detect MM CTCs. Using this technology, MM CTCs were isolated from 68% of MM patients [35–37]. However, this enrichment-based approach would miss MM CTCs that are CD138^neg^, which have been shown to possess stem cell-like qualities and a higher clonogenic potential than their CD138^pos^ counterparts [38–41].

To overcome the limitations of BM biopsies and enrichment-based CTC detection methods, we aimed to develop an assay that can detect all MM CTC subtypes, is flexible for downstream molecular characterization and, at the same time, can provide high-content morphological information. Here we describe an enrichment-free MM specific CTC assay based on the Epic Platform, which has recently been analytically validated for CTCs of epithelial origin [42]. This platform has demonstrated increased sensitivity over the CellSearch® method [43–45], and has been tested in a variety of solid tumour indications including non-small cell lung, prostate, bladder, pancreatic, ovarian and breast cancers [45–47].

The MM CTCs assay used a combination of CD138 and CD45 antibodies as well as a DAPI nuclear stain and morphology. Although CD138 has been shown to be highly expressed on most MM CTCs and is required for myeloma cell adhesion [48, 49], a clinically significant population of MM CTCs that express little to no CD138 has been described [38–41]. To increase sensitivity and ensure identification of the greater population of MM CTCs, including CD138^neg^ CTCs, we combined CD138 expression with morphology characteristics and a secondary biomarker. Due to the importance of the PI3K/AKT pathway in MM, we elected to multiplex the MM CTC assay with a phospho-ribosomal protein S6 (pS6), a common downstream readout in PI3K/AKT signalling pathway studies [50]. This approach provides a platform for enumerating and characterizing CTC in MM patients.

## 2. Methods

### 2.1 Sample receipt, processing, and CTC detection using the epic platform

**Figure 1.**
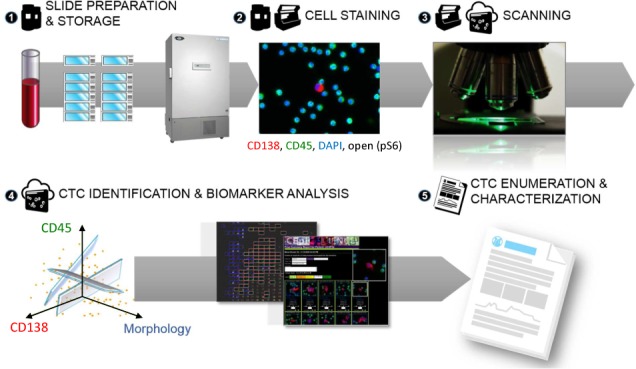
Epic Platform workflow for sample preparation, CTC enumeration and biomarker analysis. On patient blood sample receipt at Epic Sciences, (1) whole blood is lysed and nucleated cells (3 × 106 per slide) are deposited on to each of 10–12 microscope slides and stored at −80 °C until analysis. (2) Two slides per patient sample are thawed and cells are immunofluorescently labelled for CD138, CD45 and one additional biomarker, such as pS6. Nuclei are stained with DAPI. (3) Slides are scanned by a high-speed fluorescent imaging system. (4) CTC and CTC subtypes are detected and biomarker expression is quantitated utilizing Epic's proprietary digital pathology software. All CTC and CTC subtypes are confirmed by a trained human technician. (5) CTC enumeration and biomarker expression results are compiled and reported. This figure is adapted from [42].

Blood samples were collected in 10 mL cell-free DNA preservative blood tubes (Streck, Omaha, NE) and shipped to Epic Sciences for processing. Sample processing and slide preparation procedures were described previously [42, 44] and also summarized in Figure 1. Briefly, red blood cell (RBC) lysis was performed using an ammonium chloride-based buffer. Following centrifugation, all nucleated cells were plated on up to 12 glass slides at a concentration of three million nucleated cells per slide. Slides were then frozen at −80 °C until CTC analysis. On testing, two slides were thawed and immunofluorescently stained with an antibody cocktail targeting CD138 (BD Pharmingen, CA), CD45, pS6; nuclei were visualized with 4′,6-Diamidino-2-phenylindole dihydrochloride (DAPI, ThermoFisher). Slides were scanned using a high-speed fluorescent imaging system. A proprietary digital pathology algorithm identified candidate CTCs that were then reviewed by trained technicians to classify CTCs [42] into one of the following categories:

- **Traditional MM CTCs**: CD138^pos^/CD45^neg^ cells with intact, DAPI stained nuclei and morphologically distinct from surrounding white blood cells (WBCs)
- **MM CTC clusters**: two or more adjacent CTCs with shared cytoplasmic boundaries, containing at least one traditional MM CTC
- **CD138^neg^ MM CTCs**: CD138^neg^/CD45^neg^ cells with intact DAPI nuclei and morphologically distinct from surrounding WBCs
- **Apoptotic CTCs**: CD138^pos^/CD45^neg^ cells with DAPI pattern of chromosomal condensation/fragmentation and/or membrane blebbing.

The number of CTCs detected was reported as either CTC/slide or CTC/ml of blood. Data analysis and graphing were performed using Excel (Microsoft) and Prism software (GraphPad).

### 2.2 Assay development and qualification of MM CTC assay

For assay development and qualification, cell line cell (CLC) control slides were prepared following standard sample preparation procedures described above using healthy donor (HD) samples spiked with appropriate numbers of the immunoglobulin A lambda myeloma-derived, MM.1S cells. MM.1S cells were purchased from ATCC (Manassas, VA) and cultured in RPMI-1640 media supplemented with 10% foetal bovine serum at 37 °C with 5% CO_2_.

Six concentrations of a mouse monoclonal antibody against human CD138 (BD Pharmingen, CA) were tested to determine the optimal antibody concentration ranging from 0 μg/mL to 10 μg/mL. Fixation and permeabilization conditions were also optimized to obtain best signal-to-noise ratios. To determine the specificity of the anti-CD138 antibodies, control slides were stained with antibody cocktails with either the anti-CD138 antibody omitted (no primary control) or substituted with an appropriate isotype control antibody (isotype control).

For assay qualification, serial dilutions of MM.1S cells were spiked-in to HD WBCs at 1, 10, 100 and 1000 CLCs per three million WBCs per slide. Three sets of three slides at each CLC to WBC ratio listed above were prepared. The slides were stained in triplicate to determine repeatability and on three separate days to determine reproducibility. Assay sensitivity, accuracy and linearity were determined by plotting number of expected CLCs against number of recovered CLCs. In addition, assay specificity was assessed by staining one slide each of HD WBC-only samples from five individual healthy donors.

### 2.3 Patient feasibility of CTC detection in MM patients

Blood samples from three Stage III MM patients (on active treatment) were sourced from Conversant Bio (Huntsville, AL), collected in EDTA (BD Vacutainer®) tubes and shipped overnight to Epic Sciences for processing. Standard sample processing procedures were followed except blood was lysed using BD Phosflow™ Lyse/Fix Buffer 5X (BD Biosciences, San Diego, CA), and two slides from each patient were immunofluorescently labelled with an antibody cocktail against CD138, CD45, pS6 and stained with DAPI for MM CTC analysis. Following staining, CTCs were identified using Epic's proprietary algorithm as described above, and expression levels of CD138 and pS6 were measured.

## 3. Results

### 3.1 CD138 MM CTC assay development

**Figure 2.**
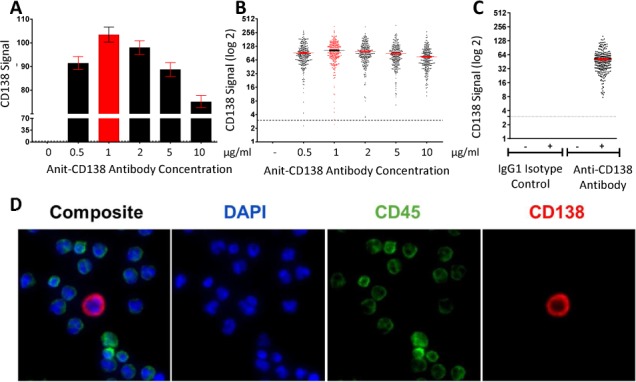
Anti-CD138 immunofluorescence-based assay was developed to detect MM CTCs. Antibody titration was performed using a monoclonal IgG1 anti-CD138 antibody on samples prepared from MM.1S cells spiked into healthy donor blood. Bar graph (A) shows mean CD138 signal ± SEM and scatter plot. (B) shows CD138 signal on each individual MM.1S cell detected. The red bar and dots denote the optimal antibody concentration chosen for the assay. The dotted line indicates the system's cut-off value. (C) Staining the same samples with the isotype control antibody or with 0 μg/ml primary antibody concentration (identified by -) resulted in no MM. 1S cells detected, confirming the specificity of the antibody staining. (D) Representative images (40x magnification) of an MM.1S cell stained with the MM CTC assay (DAPI, CD45, CD138). Note that MM.1S cells are CD138^pos^, CD45^neg^ and DAPI^pos^, whereas WBCs are CD138^neg^, CD45^pos^ and DAPI^pos^.

The anti-CD138 antibody was titrated on samples prepared from spiking a MM cell line, MM.1S, into HD WBCs, and the optimal antibody concentration providing the highest signal levels for the MM CTC assay was determined to be 1 μg/mL (Figure 2A). Although MM.1S cells represented a clonal population, a wide range of CD138 expression was observed (Figure 2B), which likely reflects the expected CD138 expression range in patient cell populations. To assess the specificity of the assay, we used no primary antibody control, where the anti-CD138 antibody was omitted, and an isotype control, where the anti-CD138 antibody was substituted with an appropriate isotype control antibody. As expected, neither of the controls detected any CD138^pos^/CD45^neg^ cells (Figures 2A, 2B, and Figure 2C), indicating the anti-CD138 antibody was specific. Representative images of an MM.1S cell with an irregularly shaped nucleus and small cytoplasm stained with the MM CTC assay (Figure 2D), showed strong and specific membrane-localized CD138 signals and absence of CD45 signals on the MM.1S cell, while the surrounding WBCs were CD45^pos^/CD138^neg^.

### 3.2 CD138 MM CTC assay qualification: Specificity, sensitivity, linearity, accuracy, repeatability and reproducibility

**Figure 3.**
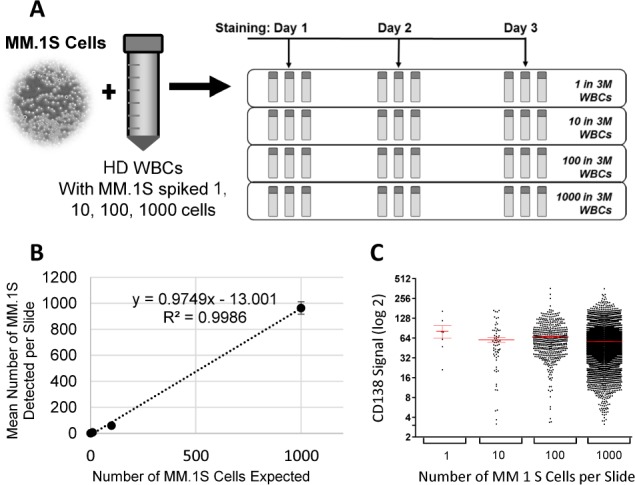
The Epic MM CTC assay is sensitive, specific, linear, accurate, repeatable and reproducible. (A) Experimental scheme for assay characterization is shown. Sample slides were prepared with a 10-fold titration series of 1, 10, 100, and 1000 MM.1S cells spiked into three million (3M) HD WBCs per slide. Triplicate slides from each titration point were stained with the MM CTC assay on three separate days. (B) The MM CTC assay is sensitive (limit of detection down to one cell), linear (R_2_=0.9986), with an accuracy of 97.5% (derived from the slope of the line). The graph shows mean ± SD numbers of MM.1S cells detected plotted against the number of MM.1S cells expected. (C) Heterogeneity of CD138 signals (mean ± SEM) were observed in MM.1S cells, but profiles of CD138 signals remained consistent regardless of number of MM.1S cells detected. The data were combined from the total of nine replicates (triplicate slides, three runs).

To qualify the MM CTC assay for patient sample analysis, assay specificity, sensitivity, linearity, accuracy, reproducibility and repeatability were evaluated. A schematic of the assay qualification setup is shown in Figure 3A. To further confirm the specificity of the MM CTC assay (in addition to the no primary and isotype controls described above), the assay was tested on WBCs isolated from five individual healthy donors and no CD138 positive cells were detected (data not shown).

**Table 1.** The MM CTC assay is repeatable across titration points. Intra-assay variability was calculated for each titration point from triplicates stained on three separate days. High consistency within the assay (low intra-assay variability, % CV<20) was demonstrated for most conditions. High % CV for n = 1 and 10 was expected due to high variability for detecting low number of cells.

Run	Expected	Mean	St dev	% CV
1	1	1	0.6	43.3
	10	9	1.5	17.6
	100	62	9.1	14.7
	1000	994	26.1	2.6
2	1	1	1.2	173.2
	10	5	0.6	10.8
	100	57	5.7	10.0
	1000	948	46.2	4.9
3	1	0	0.6	173.2
	10	7	3.5	49.5
	100	59	8.7	14.9
	1000	951	23.3	2.5

Assay sensitivity was measured by the ability of the MM CTC assay to detect down to a single MM.1S cell spiked-in to three million HD WBCs on a single slide. The results confirmed the assay is highly sensitive and validated its cut-off value. On average, one MM.1S cell per slide was detected in slides at the lowest titration point, with a range of 0 to 2 MM.1S cells detected per slide (Table 2). Furthermore, the assay showed excellent linear correlation (R^2^=0.9986) and an accuracy of 97.5% between number of expected cells and number of recovered cells ranging from 1 to 1000 cells (Figure 3B). These results also suggest the assay is unbiased since the CD138 expression profile for the CLC population remained consistent regardless of the total number of cells detected, and similar to that observed previously (Figure 3C).

**Table 2.** The MM CTC assay is reproducible across titration points. Inter-assay variability was calculated from data for each titration point across the three runs. High consistency between assay runs (low inter-assay variability, % CV<20) was demonstrated for most data points. High % CV for n = 1 and 10 was expected due to high variability for detecting low number of cells.

Expected	Mean	St dev	% CV	Range
1	1	0.8	107.2	0–2
10	7	2.4	34.3	3–10
100	59	7.3	12.3	49–72
1000	964	36.5	3.8	909–1019

Repeatability (intra-assay variability) of the MM CTC assay was determined by staining triplicate slides per titration point, while reproducibility (inter-assay variability) of the assay was determined by staining three sets of slides on three separate days. The results showed that the assay is highly repeatable and reproducible. At each titration (except for the lowest titration points, as expected), the intra-assay variability expressed as % CV between the triplicate slides (Table 1), and the inter-assay variability expressed as % CV between three runs (Table 2) were below 20%.

Taken together, these results demonstrated that the MM CTC assay was specific, sensitive, linear, accurate, unbiased, repeatable and reproducible for detecting MM-derived CLCs.

### 3.3 Patient feasibility studies using CD138 MM CTC assay

To determine the clinical feasibility of the MM CTC assay, we tested blood samples from three Stage III MM patients undergoing active treatment. Since previous work demonstrated the utility of pS6 in cytometry-based multiple myeloma CTC assays [50] and because the addition of an extra MM biomarker could potentially help identify a subpopulation of CD138^neg^/CD45^neg^ MM CTCs, we multiplexed the MM CTC assay to include pS6 as a marker for PI3K/AKT pathway activation.

**Figure 4.**
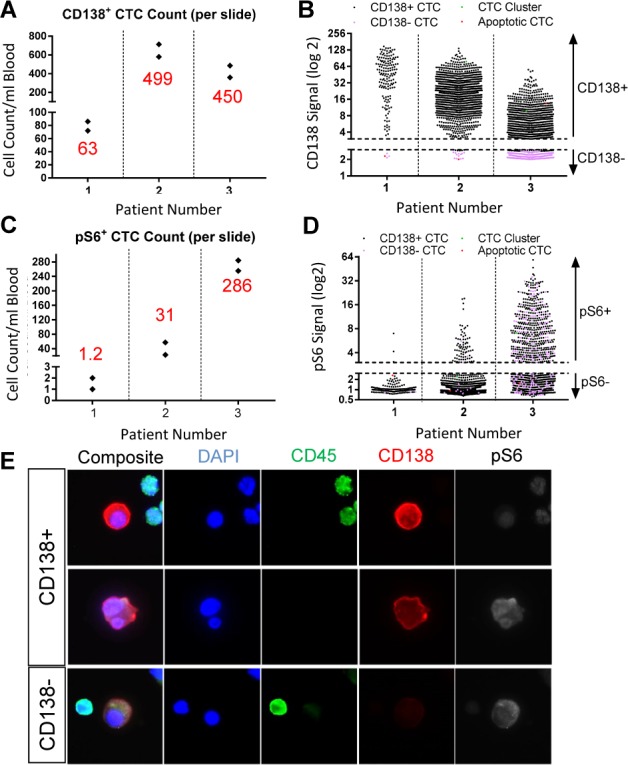
Clinical feasibility of the Epic MM CTC assay was successfully demonstrated using MM patient samples. Consistent numbers of CD138^pos^ cells (A) and pS6^pos^ cells (B) were identified on each slide within each patient sample tested (two slides were tested per patient sample and each black diamond represents cell count on one slide; red number denotes the average number of CD138^pos^ or pS6^pos^ CTCs/ml of blood). Patient CTCs also showed heterogeneous CD138 (C) and pS6 (D) signal levels (combined data from both slides per sample are shown; dotted lines indicate the negative cut-off values for CD138 and pS6 signals). (E) Representative images (40 × magnification) highlight the morphological characteristics of patient MM CTCs consistent with their plasma cell origin, such as eccentric nuclei and double-nucleated cells. Note the heterogeneity of patient MM CTCs: some were CD138^pos^/pS6^neg^ (E, top panel), some were CD138^pos^/pS6^pos^ (E, middle panel) and some were CD138^neg^/pS6^pos^ (D and E, bottom panel).

The MM CTC assay identified CD138^pos^ MM CTCs in all three patient samples tested (Figures 4A, 4B, Table 3), confirming the assay's ability to detect MM CTCs in clinical samples. Average CD138^pos^ MM CTC counts of 63 CTCs/ml, 499 CTCs/ml and 450 CTCs/ml were identified in samples from patients one, two and three, respectively. Notably, each patient sample displayed a heterogeneous population of CTCs with varying CD138 levels (Figure 4B). In addition to traditional MM CTCs, CD138^pos^ CTCs, apoptotic CTCs (characterized by abnormal nuclear morphologies, such as chromosomal condensation and/or nuclear fragmentation) and CTC clusters (defined as two or more touching CTCs) were also detected in these samples (Table 3).

**Table 3.** CTC populations in patient samples. The table summarizes the total number of CTCs from each category identified on two slides per patient. The percentage of total CTC count is reported in parentheses.

CTC category (all CD45^neg^)		Patient 1	Patient 2	Patient 3
CD138^pos^ CTCs	Traditional CTCs	157	1289	841
	CTC clusters	0	1	2
	Apoptotic CTCs	1	1	1
	CD138^pos^/pS6^pos^	3 (1.9%)	77 (6.0%)	421 (43.8%)
	CD138^pos^/pS6^neg^	155 (98.1%)	1,214 (93.8%)	423 (44.0%)
CD138^neg^ CTCs	CD138^neg^/pS6^pos^	0 (0%)	3 (0.2%)	118 (12.2%)
Total CTCs (CD138^pos^ and CD138^neg^)		158 (100%)	1294 (100%)	962 (100%)

By multiplexing the MM CTC assay with pS6 detection, we were also able to identify MM CTCs with varying levels of pS6 in all three patients' samples (Figures 4A and 4D, Table 3). Interestingly, a subpopulation of CD138^neg^/pS6^pos^ ‘putative’ CTCs were identified in samples from patients two and three (Figure 4D). Patient three demonstrated the largest subpopulation of CD138^neg^/pS6^pos^ ‘putative’ CTCs (59 CD138^neg^/pS6^pos^ CTCs out of 286 pS6^pos^ CTCs per mL of blood). These CD138^neg^/pS6^pos^ cells were classified as ‘putative’ MM CTCs based on CD45 negativity and distinct morphology from surrounding WBCs (Figure 4E), including the presence of eccentric nuclei, which is a common feature of MM cells [51].

## 4. Discussion

In this study, we developed a slide-based, enrichment-free immunofluorescence assay using the Epic Platform to detect, enumerate and characterize MM CTCs. This assay utilized CD138 and CD45 expression levels as well as morphological parameters to distinguish MM CTCs from normal WBCs. Using a titration series of MM.1S cells spiked into normal blood, we demonstrated the assay to be linear and accurate. The assay can also be multiplexed with one or two additional biomarkers to further characterize MM CTCs (discussed below).

Patient feasibility of the MM CTC assay was established by testing three patient samples, where MM CTCs were identified in all three patients ranging from 63 CTCs/mL to 498 CTCs/mL and pS6 expression levels were quantitated. Patient MM CTCs exhibited a wide range of CD138 expression levels similar to that observed in MM.1S cells. The intra- and inter-patient heterogeneity of MM CTCs was also exemplified by varying cellular morphologies as well as pS6 signal levels. Importantly, in addition to CD138^pos^/CD45^neg^ MM CTCs, a population of CD138^neg^/pS6^pos^/CD45^neg^ cells was also identified based on their pS6 expression and distinct MM-like cellular morphology. CD138^neg^ ‘putative’ MM CTCs are of particular interest as they have previously been reported to possess increased proliferative potential and correlate with poor prognosis [38–41]. A further investigation is needed to analyse characterization and confirmation of those ‘putative’ CTCs.

Basic understanding of the significance of MM CTCs and how they differ from clonal MM cells in the BM has been hampered by the lack of a sensitive and unbiased method to molecularly characterize these cells. Others have endeavoured to profile MM CTCs by capturing, culturing and comparing these cells to their resident BM counterparts [25]. However, this type of approach could introduce additional complexity due to *ex vivo* culturing of the MM CTCs. The multiplexing and downstream cell-picking capabilities of the Epic platform allow for biomarker expression and genomic profiling using the MM CTC assay described here with single-cell resolution in their native environment, providing a unique opportunity to understand disease heterogeneity.

The ability to assess and characterize MM CTCs also opens up new avenues for improving patient disease management. The invasiveness of BM biopsies limits their clinical utility typically to time of diagnosis or at disease progression. While they have been invaluable for diagnosis and for stratifying patients into proper treatment groups and clinical studies, they provide just a snapshot of a patient's disease state and are not feasible for continuous routine observations. Blood tests, on the other hand, are minimally invasive and are already performed at follow-up visits for routine disease monitoring. Adding a blood-based MM CTC assay to the current standard of care could present physicians with a movie rather than a snapshot of patients' disease evolution in real time, allowing for monitoring of treatment response, emergence of treatment resistance, appearance of potential new molecular targets and informing treatment selection.

Due to the heterogeneous nature of patient CTCs, a method capable of unbiased detection and analysis of all CTCs is required to ensure accurate assessment of a patient's disease state. The MM CTC assay described here provides an advantage over enrichment-based technologies, which are inherently incapable of detecting the full array of CTCs within each patient. Importantly, in addition to CD138^pos^/CD45^neg^ MM CTCs, in two of the three patients a clinically important population of CD138^neg^/pS6^pos^/CD45^neg^ cells was also detected, based on their pS6 expression and distinct MM-like cellular morphology. This approach may prove useful clinically for pharmacodynamic testing in new therapeutics development and for monitoring and characterizing an individual MM patient's disease via liquid biopsies of the blood.

## 6. Abbreviations

(CTC): Circulating Tumour Cell
(CLC): Cell Line Cell
(WBC): White Blood Cell
(MM): Multiple Myeloma
(BM): Bone Marrow
(HD): Healthy Donor
(HD WBCs): Healthy Donor White Blood Cells
(pS6): Phospho-ribosomal protein S6

## References

[1] Palumbo A., Role of consolidation/maintenance therapy in multiple myeloma. Clin Lymphoma Myeloma Leuk, 2013 13 Suppl 2: p. S349–54. 24290220 10.1016/j.clml.2013.05.009

[2] Palumbo A. Anderson K., Multiple myeloma. N Engl J Med, 2011 364(11): p. 1046–60. 21410373 10.1056/NEJMra1011442

[3] Ye X., Maintenance therapy with immunomodulatory drugs after autologous stem cell transplantation in patients with multiple myeloma: a meta-analysis of randomized controlled trials. PLoS One, 2013 8(8): p. e72635. 23977334 10.1371/journal.pone.0072635 PMC3747129

[4] Pineda-Roman M., VTD combination therapy with bortezomib–thalidomide–dexamethasone is highly effective in advanced and refractory multiple myeloma. Leukemia, 2008 22(7): p. 1419–1427. 18432260 10.1038/leu.2008.99 PMC3664925

[5] Nishihori T., An open-label phase I/II study of cyclophosphamide, bortezomib, pegylated liposomal doxorubicin, and dexamethasone in newly diagnosed myeloma. Eur J Haematol, 2015. 10.1111/ejh.12509 PMC4508238 25600676

[6] Kouroukis T.C., Bortezomib in multiple myeloma: systematic review and clinical considerations. Curr Oncol, 2014 21(4): p. e573–603. 25089109 10.3747/co.21.1798 PMC4117625

[7] Jeong T.D., Simplified flow cytometric immunophenotyping panel for multiple myeloma, CD56/CD19/CD138(CD38)/CD45, to differentiate neoplastic myeloma cells from reactive plasma cells. Korean J Hematol, 2012 47(4): p. 260–6. 23320004 10.5045/kjh.2012.47.4.260 PMC3538797

[8] Lonial S. Anderson K.C., Association of response endpoints with survival outcomes in multiple myeloma. Leukemia, 2014 28(2): p. 258–268. 23868105 10.1038/leu.2013.220 PMC3918869

[9] Harousseau J.-L., Better quality of response to lenalidomide plus dexamethasone is associated with improved clinical outcomes in patients with relapsed or refractory multiple myeloma. Haematologica, 2010 95(10): p. 1738–1744. 20460639 10.3324/haematol.2009.015917 PMC2948100

[10] Fuhler G.M., Widespread deregulation of phosphorylation-based signaling pathways in multiple myeloma cells: opportunities for therapeutic intervention. Mol Med, 2011 17(7–8): p. 790–8. 21541441 10.2119/molmed.2011.00013 PMC3146594

[11] Younes H., Targeting the phosphatidylinositol 3-kinase pathway in multiple myeloma. Clin Cancer Res, 2007 13(13): p. 3771–5. 17606706 10.1158/1078-0432.CCR-06-2921

[12] Munugalavadla V., The PI3K inhibitor GDC-0941 combines with existing clinical regimens for superior activity in multiple myeloma. Oncogene, 2014 33(3): p. 316–25. 23318440 10.1038/onc.2012.594

[13] Steinbrunn T., Combined targeting of MEK/MAPK and PI3K/Akt signalling in multiple myeloma. Br J Haematol, 2012 159(4): p. 430–40. 22985491 10.1111/bjh.12039

[14] Baumann P., Simultaneous targeting of PI3K and mTOR with NVP-BGT226 is highly effective in multiple myeloma. Anticancer Drugs, 2012 23(1): p. 131–8. 21959532 10.1097/CAD.0b013e32834c8683

[15] Ikeda H., PI3K/p110{delta} is a novel therapeutic target in multiple myeloma. Blood, 2010 116(9): p. 1460–8. 20505158 10.1182/blood-2009-06-222943 PMC2938837

[16] Kandoth C., Mutational landscape and significance across 12 major cancer types. Nature, 2013 502(7471): p. 333–9. 24132290 10.1038/nature12634 PMC3927368

[17] de Bruin E.C., Spatial and temporal diversity in genomic instability processes defines lung cancer evolution. Science, 2014 346(6206): p. 251–6. 25301630 10.1126/science.1253462 PMC4636050

[18] Burrell R.A., The causes and consequences of genetic heterogeneity in cancer evolution. Nature, 2013 501(7467): p. 338–345. 24048066 10.1038/nature12625

[19] Greaves M. Maley C.C., Clonal evolution in cancer. Nature, 2012 481(7381): p. 306–13. 22258609 10.1038/nature10762 PMC3367003

[20] Hiley C., Deciphering intratumor heterogeneity and temporal acquisition of driver events to refine precision medicine. Genome Biol, 2014 15(8): p. 453. 25222836 10.1186/s13059-014-0453-8 PMC4281956

[21] Barber L.J. Davies M.N. Gerlinger M., Dissecting cancer evolution at the macro-heterogeneity and micro-heterogeneity scale. Curr Opin Genet Dev, 2014 30c: p. 1–6. 10.1016/j.gde.2014.12.001 PMC4728189 25555261

[22] Carreira S., Tumor clone dynamics in lethal prostate cancer. Sci Transl Med, 2014 6(254): p. 254ra125. 10.1126/scitranslmed.3009448 PMC4422178 25232177

[23] Mateo J., The promise of circulating tumor cell analysis in cancer management. Genome Biol, 2014 15(8): p. 448. 25222379 10.1186/s13059-014-0448-5 PMC4281949

[24] Crowley E., Liquid biopsy: monitoring cancergenetics in the blood. Nat Rev Clin Oncol, 2013 10(8): p. 472–84. 23836314 10.1038/nrclinonc.2013.110

[25] Paiva B., Detailed characterization of multiple myeloma circulating tumor cells shows unique phenotypic, cytogenetic, functional, and circadian distribution profile. Blood, 2013 122(22): p. 3591–8. 24072855 10.1182/blood-2013-06-510453

[26] Witzig T.E., Quantitation of circulating peripheral blood plasma cells and their relationship to disease activity in patients with multiple myeloma. Cancer, 1993 72(1): p. 108–13. 8508395 10.1002/1097-0142(19930701)72:1<108::aid-cncr2820720121>3.0.co;2-t

[27] Rawstron A.C., Circulating plasma cells in multiple myeloma: characterization and correlation with disease stage. Br J Haematol, 1997 97(1): p. 46–55. 9136941 10.1046/j.1365-2141.1997.72653.x

[28] Dingli D., Flow cytometric detection of circulating myeloma cells before transplantation in patients with multiple myeloma: a simple risk stratification system. Blood, 2006 107(8): p. 3384–8. 16339399 10.1182/blood-2005-08-3398 PMC1895764

[29] Gertz M.A., Monoclonal plasma cells in the blood stem cell harvest from patients with multiple myeloma are associated with shortened relapse-free survival after transplantation. Bone Marrow Transplant, 1997 19(4): p. 337–42. 9051243 10.1038/sj.bmt.1700670

[30] Nowakowski G.S., Circulating plasma cells detected by flow cytometry as a predictor of survival in 302 patients with newly diagnosed multiple myeloma. Blood, 2005 106(7): p. 2276–9. 15961515 10.1182/blood-2005-05-1858 PMC1895270

[31] Paiva B. van Dongen J.J. Orfao A., New criteria for response assessment: role of minimal residual disease in multiple myeloma. Blood, 2015 125(20): p. 3059–68. 25838346 10.1182/blood-2014-11-568907 PMC4513329

[32] Billadeau D., Clonal circulating cells are common in plasma cell proliferative disorders: a comparison of monoclonal gammopathy of undetermined significance, smoldering multiple myeloma, and active myeloma. Vol. 88 1996 289–296. 8704185

[33] Luque R., Normal and clonal B lineage cells can be distinguished by their differential expression of B cell antigens and adhesion molecules in peripheral blood from multiple myeloma (MM) patients—diagnostic and clinical implications. Clinical and Experimental Immunology, 1998 112(3): p. 410–418. 9649209 10.1046/j.1365-2249.1998.00600.x PMC1904982

[34] Schneider U., Two subsets of peripheral blood plasma cells defined by differential expression of CD45 antigen. Br J Haematol, 1997 97(1): p. 56–64. 9136942 10.1046/j.1365-2141.1997.d01-2115.x

[35] Gross S., Assay to capture and detect circulating multiple myeloma cells from blood. 2013, Google Patents.

[36] Gross S. Automated Enumeration and Characterization of Circulating Multiple Myeloma Cells in Blood. in ASH Annual Meeting Abstracts. 2011.

[37] Weiss B., Circulating Multiple Myeloma Cells (CMMCs): A Novel Method for Detection and Molecular Characterization of Peripheral Blood Plasma Cells in Multiple Myeloma Precursor States. Blood, 2014 124(21): p. 2031–2031.

[38] Reid S., Characterisation and relevance of CD138-negative plasma cells in plasma cell myeloma. Int J Lab Hematol, 2010 32(6 Pt 1): p. e190–6. 20201998 10.1111/j.1751-553X.2010.01222.x

[39] Matsui W., Characterization of clonogenic multiple myeloma cells. Blood, 2004 103(6): p. 2332–2336. 14630803 10.1182/blood-2003-09-3064 PMC3311914

[40] Jacak J., Expression analysis of multiple myeloma CD138 negative progenitor cells using single molecule microarray readout. Journal of Biotechnology, 2013 164(4): p. 525–530. 23416329 10.1016/j.jbiotec.2013.01.027 PMC3632753

[41] Kawano Y., Multiple myeloma cells expressing low levels of CD138 have an immature phenotype and reduced sensitivity to lenalidomide. Int J Oncol, 2012 41(3): p. 876–84. 22766978 10.3892/ijo.2012.1545 PMC3582943

[42] Werner S.L., Analytical Validation and Capabilities of the Epic CTC Platform: Enrichment-Free Circulating Tumour Cell Detection and Characterization. J Circ Biomark, 2015 4(3): p. doi: 10.5772/60725. 10.5772/60725 PMC5572988 28936239

[43] Dittamore R. Molecular characterization of circulating tumor cells (CTC) and CTC subpopulations in baseline and progressive metastatic castration resistant prostate cancer (mCRPC). in ASCO Annual Meeting Proceedings. 2014.

[44] Marrinucci D., Fluid biopsy in patients with metastatic prostate, pancreatic and breast cancers. Phys Biol, 2012 9(1): p. 016003. 22306768 10.1088/1478-3975/9/1/016003 PMC3387996

[45] Nieva J., High-definition imaging of circulating tumor cells and associated cellular events in non-small cell lung cancer patients: a longitudinal analysis. Phys Biol, 2012 9(1): p. 016004. 22306961 10.1088/1478-3975/9/1/016004 PMC3388002

[46] Punnoose E., PTEN loss in Circulating Tumor Cells (CTCs) correlates with PTEN loss in fresh tumor tissue from Castration-Resistant Prostate Cancer (CRPC) patients. Br J Cancer, 2015(submitted). 10.1038/bjc.2015.332 PMC4647881 26379078

[47] Scher H.I., Predictive biomarkers of sensitivity to androgen receptor signaling (ARS) and taxane-based chemotherapy in circulating tumor cells (CTCs) of patients (pts) with metastatic castration resistant prostate cancer (mCRPC). J Clin Oncol, 2015 33(suppl 7, abstr 147).

[48] Khotskaya Y.B., Syndecan-1 Is Required for Robust Growth, Vascularization, and Metastasis of Myeloma Tumors in Vivo. Journal of Biological Chemistry, 2009 284(38): p. 26085–26095. 19596856 10.1074/jbc.M109.018473 PMC2758008

[49] Kumar S. Kimlinger T. Morice W., Immunophenotyping in multiple myeloma and related plasma cell disorders. Best practice & research. Clinical haematology, 2010 23(3): p. 433–451. 21112041 10.1016/j.beha.2010.09.002 PMC3005703

[50] Li C., Development of a robust flow cytometry-based pharmacodynamic assay to detect phospho-protein signals for phosphatidylinositol 3-kinase inhibitors in multiple myeloma. J Transl Med, 2013 11: p. 76. 23522020 10.1186/1479-5876-11-76 PMC3623880

[51] San Miguel J.F., Conventional diagnostics in multiple myeloma. Eur J Cancer, 2006 42(11): p. 1510–9. 16762540 10.1016/j.ejca.2005.11.039

